# Electrochemical Approximation to Bronze Age Chronology via Multiple Scan Voltammetry

**DOI:** 10.1002/celc.202300405

**Published:** 2023-11-06

**Authors:** Antonio Doménech‐Carbó, Marianne Mödlinger, Laura Osete‐Cortina, María Teresa Doménech‐Carbó

**Affiliations:** ^1^ Department of analytical chemistry Universitat de València Dr. Moliner, 50 46100 Burjassot València Spain; ^2^ Institut für Archäologien Universität Innsbruck Langer Weg 11 6020 Innsbruck Austria; ^3^ Institut für Archäologien Universität Innsbruck Langer Weg 11 6020 Innsbruck Austria; ^4^ Institut de Restauració del Patrimoni Universitat Politècnica de València Camí de Vera, 14 46022 València Spain

**Keywords:** Archaeology, Bronze, Dating, Electrochemistry

## Abstract

Insert A multiple‐scan voltammetry strategy is described and applied to a set of 107 Bronze Age and later copper/bronze objects, mainly from sites in Central Europe. This methodology allows the study of the compositional and textural properties (compactness, crystallinity, degree of hydration) of the patina to be studied from the accumulated peak current values for the characteristic signals corresponding to the reduction of cuprite and tenorite to metallic copper. A new model for the relationship between peak current and the depth reached in successive scans is presented and used to discriminate samples of different provenance and manufacturing technique, as well as their ascription to different Bronze Age periods

## Graphical abstract

**Electrochemistry in archaeology**: The chronology of a series of bronze Age objects from central Europe was assessed using multiple‐scan solid state voltammetry.

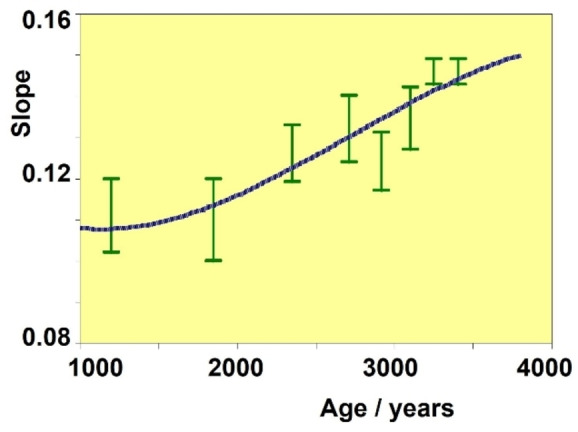

## Introduction

The study of archaeological metal objects presents considerable difficulties due to their great diversity in composition, microstructure and corrosion conditions, which are related to many different factors such as the chemical composition of the object itself and the context in which it was found, i. e. aerobic or anaerobic conditions, soil, (salt)water, and more.^[1]^ Obtaining chronological information on archaeological metals with a higher precision than provided by archaeological typological classification or the study of the archaeological context is an important analytical goal.^[2]^ This is usually achieved by dating associated organic matter, if available, via radiocarbon methods, and/or associated ceramic materials by thermoluminescence, obsidian hydration, and rehydroxylation methods. However, direct dating of archaeological metal finds has been virtually limited to the use of the Meissner superconducting effect for lead,^[3]^ and He, U, and Th analysis for gold.[^4^, ^5^] Both methods require the disposal of sample quantities of several hundred milligrams, thus limiting their practical application in most cases. Because each archaeological object is a unique record of our history, invasive analyses are usually not well received by museums. Sampling which damages the object is only allowed in one area per object if at all. Multiple invasive analyses of the same object are very rare. Accordingly, patina‐focused analytical methods are increasingly demanded by the archaeometric community.[^6^, ^7^, ^8^, ^9^] In this sense, electrochemical methods have contributed significantly to the understanding of corrosion phenomena[^10^, ^11^, ^12^] and a variety of proposals have recently been reported in the study of metallic heritage.[^13^, ^14^, ^15^, ^16^]

In this context is of application the voltammetry of immobilized particles (VIMP), a technique developed by Scholz et al.[^17^, ^18^, ^19^] that displays analytical information on sparingly soluble solids in contact with suitable electrolytes. Since the VIMP involves nanogram quantities of solid sample, it provides archaeometric information for authentification and discrimination of the provenance and/or manufacturing technique of metal artifacts.[^20^, ^21^, ^22^] In the last decade, we have explored the application of the VIMP for dating metals, including lead,^[23]^ leaded bronze,^[24]^ and gold.[^25^, ^26^, ^27^] In particular, we proposed a method for dating^[28]^ and tracing provenance^[29]^ of copper and bronze objects based on the voltammetric estimation of the tenorite/cuprite ratio in the patina of the artifacts. This method gives satisfactory results for series of objects with homogeneous composition and a fairly similar and smooth ‘corrosion history’, but the data scatter increases significantly when these conditions are not met.

**Figure 1:**
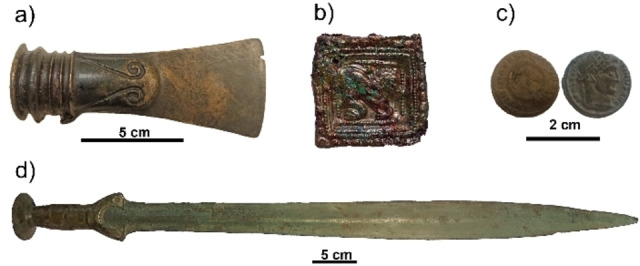
Photographic image of some of the objects analized. a) bronze axe from Sieding, Austria (isolated find, Ha B period, MAMUZ Museum, inv. No. UF‐5098), b) part of a belt from a grave at Micheldorf (Austria) (Medieval period, University of Innsbruck, inv. No. A7105), c) coin from Bad Deutsch‐Altenburg, Flur Mühläcker (Austria) (first third 4th century AD, Art History Museum (Vienna), inv. No. 9/84), and d) bronze sword from Vulchovica, Ukraine (Ha B1 period, National History Museum (Vienna), inv.no. 1926).

Here, we describe a VIMP‐based methodology consisting of multiple scan voltammetry for obtaining chronological relationships on archaeological copper/bronze objects. As in the previously reported single‐scan methodology,[^28^, ^29^] abrasive sampling provides a set of micrometric‐sized metal flakes that are adhered to the base graphite electrode and placed in contact with the electrolyte. The basic idea is that, since the composition of the metal patina is depth‐dependent, the application of successive reductive potential inputs will cause the progressive delamination of the sheets thus providing information about deeper regions of the patina.[^27^, ^30^, ^31^, ^32^] It is then hypothesized that the gradient of the tenorite/cuprite ratio (accessible by multiple‐scan voltammetry) rather than its ‘absolute’ value in the external patina (determined by single‐scan voltammetry), can be taken as an age marker suitable for constructing a calibration curve. Data from a series of 107 copper/bronze objects from the Bronze Age onwards, mainly recovered from sites in central Europe, are presented from different Austrian museums, such as the MAMUZ (Asparn an der Zaya), the University of Innsbruck (Innsbruck), as well as the Natural History and the Art History Museum (Vienna), previously studied by conventional techniques.[^33^, ^34^, ^35^, ^36^] Complementary experiments on medieval objects and modern coins already described^[31]^ are also reported. Figure 1 shows photographic images of some of the Bronze Age objects studied.

## Results and discussion

### Voltammetric pattern

**Figure 2:**
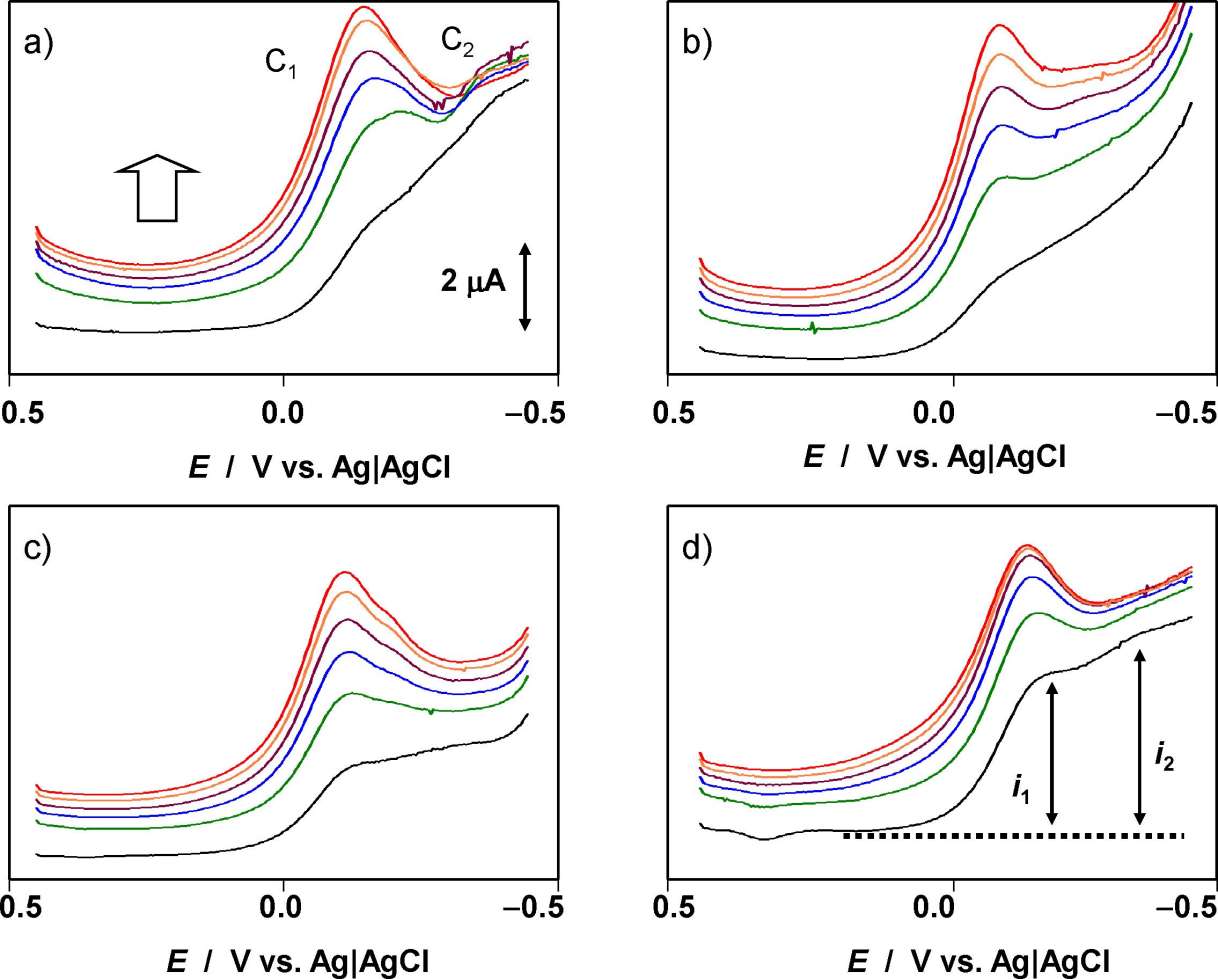
Square wave voltammetry of sample‐modified graphite electrode in contact with air‐saturated 0.25 M HAc/NaAc aqueous buffer at pH 4.75. a) Bronze sword from unknown find spot (Bz B1 period, Natural History Museum, Vienna, inv. no. 35012); b) bronze knife from Prigglitz‐Gasteil, Austria (Ha B2‐3 period, MAMUZ Museum, inv. no. UF‐10,964); c) casting cake from the Siedlungsareal Ruine Knopfsberg, Austria (Ha C period, University of Innsbruck, inv. no. K443); d) bronze dagger from Koban, Russia (Natural History Museum, Vienna, inv. no. 41283) of unknown chronology. The dotted line in d) represents the base line used to measure the peak current in the 1st scan. The gross arrow in a) indicates the evolution of the peak C_1_ in successive potential scans. Potential scan initiated at 0.45 V in the negative direction; potential step increment 4 mV; square wave amplitude 25 mV; frequency 10 Hz.

Figure 2 shows the repetitive square wave voltammetry of four archaeological samples in contact with air‐saturated 0.25 M HAc/NaAc aqueous buffer at pH 4.75. The electrolyte solution was not deoxygenated to mimic the conditions employed when portable instruments are used for field analysis. The potential was scanned from 0.45 V vs. Ag/AgCl in the negative direction, resulting in the reduction of copper corrosion products. Under moderate corrosion conditions, no green corrosion products of the malachite, brochantite and atacamite mineral families are formed and the patina is composed mainly of cuprite (Cu_2_O) and tenorite (CuO) with varying degrees of compactness and crystallinity.[^8^, ^10^] As previously described in detail,[^28^, ^29^, ^30^, ^31^, ^32^] under these experimental conditions these components exhibit reduction peaks at about −0.15 (C_1_) and −0.40 V vs. Ag/AgCl (C_2_), respectively, corresponding to their proton‐assisted reduction to copper metal. These processes can be represented as (Eqs. 1–2,
(1)

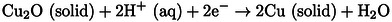



(2)

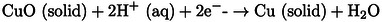




However, this second signal is superimposed by some background current associated with the reduction of dissolved oxygen. When the potential scan is subsequently reversed, an anodic peak, often with peak splitting, is recorded at ca. 0.0 V (see Supplementary Information, Figure S.1). This peak corresponds to the oxidative dissolution of the previously formed copper metal deposit to Cu^2+^ (aq) ions in the solution phase. In general, tin‐localized voltammetric signals were absent, a result of the well‐known destannification phenomenon.[^6^, ^7^, ^8^, ^9^, ^10^]

**Figure 3:**
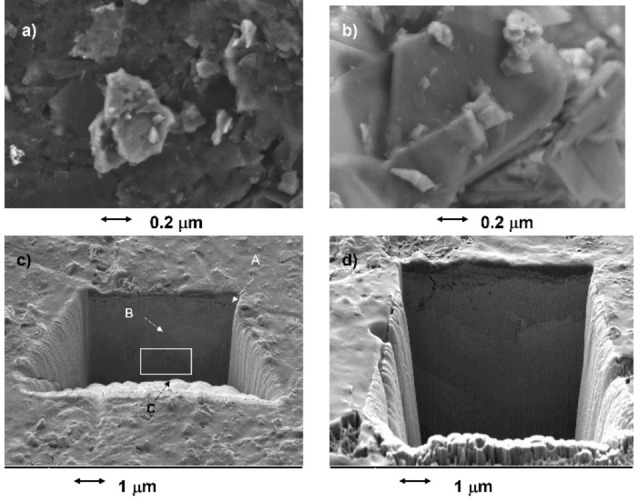
a) Secondary electron image of a graphite plate after sampling on a 5‐cent Belgium coin emitted in 1953, showing metal sheets extracted from the patina; b) detail of a metal sheet showing its laminar‐like configuration; c,d) FIB‐FESEM images of trenches on c) 10‐cent Spanish coin (Isabel II, 1868), and d) 5‐cent French coin (République Francaise, 1916), both composed of binary Cu−Sn bronze.

In the typical VIMP sampling procedure, a graphite bar is pressed across the surface of the metallic object. Depending on the pressure exerted over the surface of the artifact and the hardness and compactness of its patina, a more or less numerous set of more or less thick patina flakes is extracted and attached to the graphite bar. Typically, these are micrometric in size, as can be seen in the SEM images in Figures 3a,b. Excavation of trenches by means of fast atomic bombardment (see FIB/FESEM images in Figures 3c,d) shows that the outer region of metal objects has a stratified texture frequently with an outer layer of 1–2 μm thick. Apparently, the extracted patina flakes correspond to this external region, confirming previous studies.[^29^, ^31^, ^37^, ^38^] Ideally, high pressures will extract thicker laminae from the metal corrosion patina so that they are representative of the chemical and textural properties mentioned above in deeper regions. Then, the application of successive potential scans will cause the progressive delamination of the plates adhered to the graphite electrode thus obtaining voltammetric signals representative of increasingly deeper regions of the metal patina.[^31^, ^32^] As schematized in Figure S.2 of the Supplementary Information, since the electrochemical processes starts at the patina sheet/graphite/electrolyte three‐phase boundary,[^17^, ^18^, ^19^, ^20^] the initial cathodic processes correspond to the reduction of the components of the more external region of the metal patina. It is worth emphasizing that the net amount of sample transferred to the graphite electrodes is a few nanograms, as judged by the area under the voltammetric peaks measured in linear potential scan measurements.

### Deep analysis

**Figure 4:**
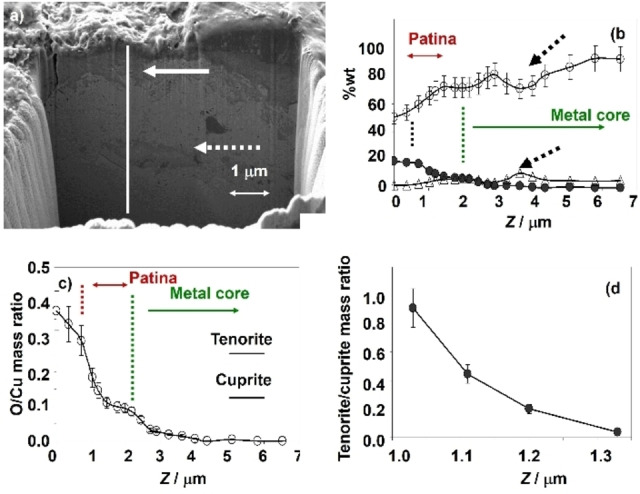
a) FIB‐FESEM image of a trench on a 95 %wt Cu plus 5 %wt Zn 100 year old coin; b) deep profiles of Cu (circles), O (solid circles), and Zn (triangles) determined by SEM/EDX; c) deep variation of the O/Cu mass ratio from the above data; d) deep variation of the tenorite/cuprite ratio in the patina calculated from SEM/EDX data. The vertical line in (a) indicates the direction in which EDX analysis was performed, the continuous arrow indicates the corrosion patina and the dotted arrow indicates the position of a Zn‐rich inclusion within the metal core region.

The previous considerations are consistent with data on the deep composition determined by SEM/EDX for copper/bronze coins.[^37^, ^38^] Figure 4 compares the deep variation of the Cu, O, and Zn contents determined by SEM/EDX in a FIB trench produced in a 95 %wt Cu plus 5 % wt Zn 100 year old coin. The SEM image of the trench (Figure 4a) shows a thin external layer covering a dark grey region 1–2 μm thick (white horizontal arrow) over the faceted inner area including an island marked by a horizontal dotted arrow. The concentration profiles of Cu, O, and Zn (Figure 4b) and the O/Cu mass ratio (Figure 4c) determined from EDX data show that the outer layer is rich in oxygen. In fact, the O/Cu mass ratio is greater than the theoretical value for tenorite, the higher expected copper oxide (CuO), 0.252. This is due, in agreement with previous studies on metal coins,^[39]^ to the presence of organic matter and S‐ and Cl‐containing inorganic salts. According to Robbiola et al.,^[10]^ this layer can be regarded as the tertiary patina, that under burial conditions is formed via pedological processes. The region between (approximately) 0.5 and 2.5 μm depth forms the ‘true’ (primary plus secondary) patina which is composed by cuprite and tenorite as judged by deep Raman spectroscopy data.^[40]^

Assuming that cuprite and tenorite are the only components of the ‘true’ patina, the SEM/EDX data allow us to estimate the tenorite/cuprite mass ratio. The deep variation of this ratio calculated from SEM/EDX can be compared with the same ratio calculated as the ratio of the peak currents measured for the signals C_1_ and C_2_ in multiple‐scan VIMP experiments under conditions such as in Figure 2, as described in the next section. The modeling presented in this section allows us to relate the depth *z* reached at the *N* ^th^ scan to the accumulated peak current at this potential scan. Figure 4d shows the depth variation of the tenorite/cuprite mass ratio calculated from SEM/EDX data. Although the VIMP data do not allow the estimation of absolute depth values, it is interesting to note that the variation of the tenorite to cuprite mass ratio in Figure 4d is close to the variation of the C_2_ to C_1_ peak current ratio with the number of scans (see below) and with the accumulated peak current (see Supplementary information, Figure S.3).

These results are consistent with the hypothesis underlying the current work: that the voltammetric parameters in repetitive voltammetry are representative of the gradient of composition, crystallinity, compactness, etc. in the corrosion patina, to the presence of organic components and S‐ and Cl‐containing inorganic salts.^[39]^

The above hypothesis is also consistent with several features already described: i) that the position and shape of voltammetric signals in VIMP experiments depends not only on the composition, but also on the shape and size of the microparticles;^[18]^ ii) detailed Raman spectroscopy data showing peaks characterizing crystalline cuprite (114 and 220 cm^‐1^), amorphous or finely divided (defective) cuprite (420, 525, 625 cm^−1^), crystalline tenorite (297 cm^−1^) and possibly less crystalline tenorite (346 and 631 cm^−1^) in copper‐based patinas;^[40]^ iii) the dependence of electrochemical impedance spectroscopy parameters, directly related to textural properties (compactness, porosity, roughness) of metal patinas,^[41]^ on sampling; iv) the agreement in the grouping of archaeological metal samples using VIMP and EIS[^37^, ^41^] and VIMP and Raman spectroscopy.[^37^, ^42^] In addition, it should be noted that the intersection of the SEM/EDX and VIMP data indicates that, under our experimental conditions, the voltammetric experiments are limited to the depth of the patina region in Figure 4. See ‘Dept calculations’ in the Supplementary information.

### Modeling

Using the above conceptual framework, the system under study can ideally be represented as a set of *k* plates of thickness *δ* and surface *A*, which are progressively delaminated during electrochemical reduction. In the case of moderately corroded objects, the patina will be composed by cuprite and tenorite whose crystallinity, compactness and porosity vary with the depth *z*. Accordingly, the density of each component will vary from a value *ρ*_inn_ in the inner, compact primary patina, to a value*ρ*_surf_ in the external surface region of the secondary patina. Under this view, the density of electroactive component, *ρ*, can be expressed as a continuous function of *z*, *ρ*(*z*), so that *z*(*N*) < *δ* represents the depth reached in the *N* ^th^ voltammetric scan. Our experimental data (see below) suggest that *ρ*(*z*) can be approximated by potential functions of the form (Eq. 3,

(3) $$\rho \left(z\right)=\rho_{\mathrm{inn}}-\left(\rho_{\mathrm{inn}}-\rho_{\mathrm{surf}}\right){\left(1-\frac{z}{z_{\mathrm{inn}}}\right)}^{\alpha }\;$$

so that when *z*=0, *ρ*(*z*)=*ρ*_surf_ and when *z*=*z*_inn_, *ρ*(*z*)=*ρ*_inn_. In this equation, *α* is an adjustable parameter whose value must be decided from experimental data. The charge passed during the first scan for a *n*‐electron process on a sheet of area *A*, *q*(1), in which a depth *z*_1_ is reached will be (Eqs. 4–5:

(4) $$q\left(1\right)=nFA\int_{z=0}^{z_{1}}[\rho_{\mathrm{inn}}-\left(\rho_{\mathrm{inn}}-\rho_{\mathrm{surf}})(1-\frac{z}{z_{\mathrm{inn}}}{)}^{\alpha }\right]dz\;$$

i. e.,

(5) $$q\left(1\right)=nFA\rho_{\mathrm{inn}}z+nFAz_{\mathrm{inn}}\left(\frac{\rho_{\mathrm{inn}}-\rho_{\mathrm{surf}}}{1+\alpha }\right)\left[{\left(1-\frac{z_{1}}{z_{\mathrm{inn}}}\right)}^{1+\alpha }-1\right]$$

Accordingly, the charge passed at the *N* ^th^ scan, when a depth *z*_N_ is reached, will be (Eq. 6,

(6) $$q\left(N\right)=nFA\rho_{\mathrm{inn}}\left(z_{N}-z_{N-1}\right)+nFAz_{\mathrm{inn}}\left(\frac{\rho_{\mathrm{inn}}-\rho_{\mathrm{surf}}}{1+\alpha }\right)\left[{\left(1-\frac{z_{N}}{z_{\mathrm{inn}}}\right)}^{1+\alpha }-{\left(1-\frac{z_{N-1}}{z_{\mathrm{inn}}}\right)}^{1+\alpha }\right]$$

It can easily be demonstrated that on computing the cumulated charge at the *N* ^th^ scan, *Q*(*N*) (=*q*(1)+*q*(2)+…+*q*(*N*)), several *z*‐terms cancel so that (Eq. 7:

(7) $$Q\left(N\right)=nFA\left[\rho_{\mathrm{inn}}z_{N}+z_{\mathrm{inn}}\left(\frac{\rho_{\mathrm{inn}}-\rho_{\mathrm{surf}}}{1+\alpha }\right)\left[{\left(1-\frac{z_{N}}{z_{\mathrm{inn}}}\right)}^{1+\alpha }-1\right]\right]\;$$

This equation predicts a potential variation of *Q*(*N*) with *z*. As a particular case, if the density of electroactive product increases linearly with depth, *α*=1 and Eq. (7) becomes (Eqs. 8–9:

(8) $$Q\left(N\right)=nFA\left[\rho_{\mathrm{inn}}z_{N}+z_{\mathrm{inn}}\left(\frac{\rho_{\mathrm{inn}}-\rho_{\mathrm{surf}}}{2}\right)\left[{\left(1-\frac{z_{N}}{z_{\mathrm{inn}}}\right)}^{2}-1\right]\right]\;$$

When *z*_N_=*z*_inn_,

(9) $$Q\left(N\right)=nFAz_{\mathrm{inn}}\left(\frac{\rho_{\mathrm{inn}}-\rho_{\mathrm{surf}}}{2}\right)$$

The above modeling can be considered as representative of the variation of the cuprite density through the secondary patina. An equivalent model can be taken for tenorite now assuming that the amount of this component decreases from the external surface to the internal region. Then, assuming a potential variation of the tenorite density, *σ*(*z*), with depth given in terms of an exponent *β*, one can take (Eq. 10,

(10) $$\sigma \left(z\right)=\sigma_{\mathrm{inn}}+\left(\sigma_{\mathrm{surf}}-\sigma_{\mathrm{inn}}\right){\left(1-\frac{z}{z_{\mathrm{inn}}}\right)}^{\beta }\;$$

where *σ*_inn_, *σ*_surf_, are the tenorite densities at the primary patina and the external surface of the same, respectively. Then, the accumulated charge passed at the *N* ^th^ potential scan will be (Eq. 11,

(11) $$Q\left(N\right)=nFA\left[\sigma_{\mathrm{inn}}z_{N}-z_{\mathrm{inn}}\left(\frac{\sigma_{\mathrm{surf}}-\sigma_{\mathrm{inn}}}{1+\beta }\right)\left[{\left(1-\frac{z_{N}}{z_{\mathrm{inn}}}\right)}^{1+\beta }-1\right]\right]\;$$

Accordingly, the ratio between the accumulated charges passed in the tenorite‐ and cuprite‐centered reduction processes, *Q*_ten_(*N*)/*Q*_cup_(*N*), can be expressed as (Eq. 12,

(12) $$\frac{Q_{\mathrm{ten}}\left(N\right)}{Q_{\mathrm{cup}}\left(N\right)}=\frac{\left[\sigma_{\mathrm{inn}}z_{N}-z_{\mathrm{inn}}\left(\frac{\sigma_{\mathrm{surf}}-\sigma_{inn}}{1+\beta }\right)\left[{\left(1-\frac{z_{N}}{z_{\mathrm{inn}}}\right)}^{1+\beta }-1\right]\right]}{\left[\rho_{\mathrm{inn}}z_{N}+z_{\mathrm{inn}}\left(\frac{\rho_{\mathrm{inn}}-\rho_{\mathrm{surf}}}{1+\alpha }\right)\left[{\left(1-\frac{z_{N}}{z_{\mathrm{inn}}}\right)}^{1+\alpha }-1\right]\right]}\;$$

The above formalism can be formulated in terms of the peak currents measured in voltammetric experiments, assuming that the (difference) square wave peak currents measured under our experimental conditions are representative of the net charge transferred in the corresponding reduction process. Assuming that the sample‐modified electrode contains *k* identical flakes, one can write (Eq. 13,

(13) $$i_{J}\;\left(N\right)=\;knh_{J}\delta \left(N\right)\rho_{J}\left(N\right)f_{J}\left(N\right)A+b_{J}\left(N\right)$$

where *i*_J_(*N*) represents the intensity of the voltammetric signal (C_1_, C_2_) corresponding to the reduction of the J‐component (cuprite, tenorite) at the *N* ^th^ potential scan, *δ*(*N*) the thickness of the *N* ^th^ layer and *ρ*_J_(*N*) the average density of the *J*‐component in the *N* ^th^ layer. *f*_J_(*N*) represents the mass fraction of the *J*‐component in the *N* ^th^ layer and *h*_J_ is an electrochemical constant characteristic of each component. The above equation includes a background term, *b*_J_(*N*), which depends on the potential at which the signal appears and the amount of scratching produced in the graphite surface during the sampling process.

Since the voltammograms are initiated at a potential (0.45 V) more positive than those at which the oxidative dissolution of copper to Cu^2+^ (aq) occurs (ca. 0.0 V, see Figure S.1 in Supplementary Information), some fraction of these species can also be reduced to copper metal in successive negative‐going potential scans. As far as this process will be superimposed to the reduction of cuprite, the successive negative‐going voltammograms will reflect the composition of successive layers of the patina in a form more complex than that represented by Eq. (13).

**Figure 5:**
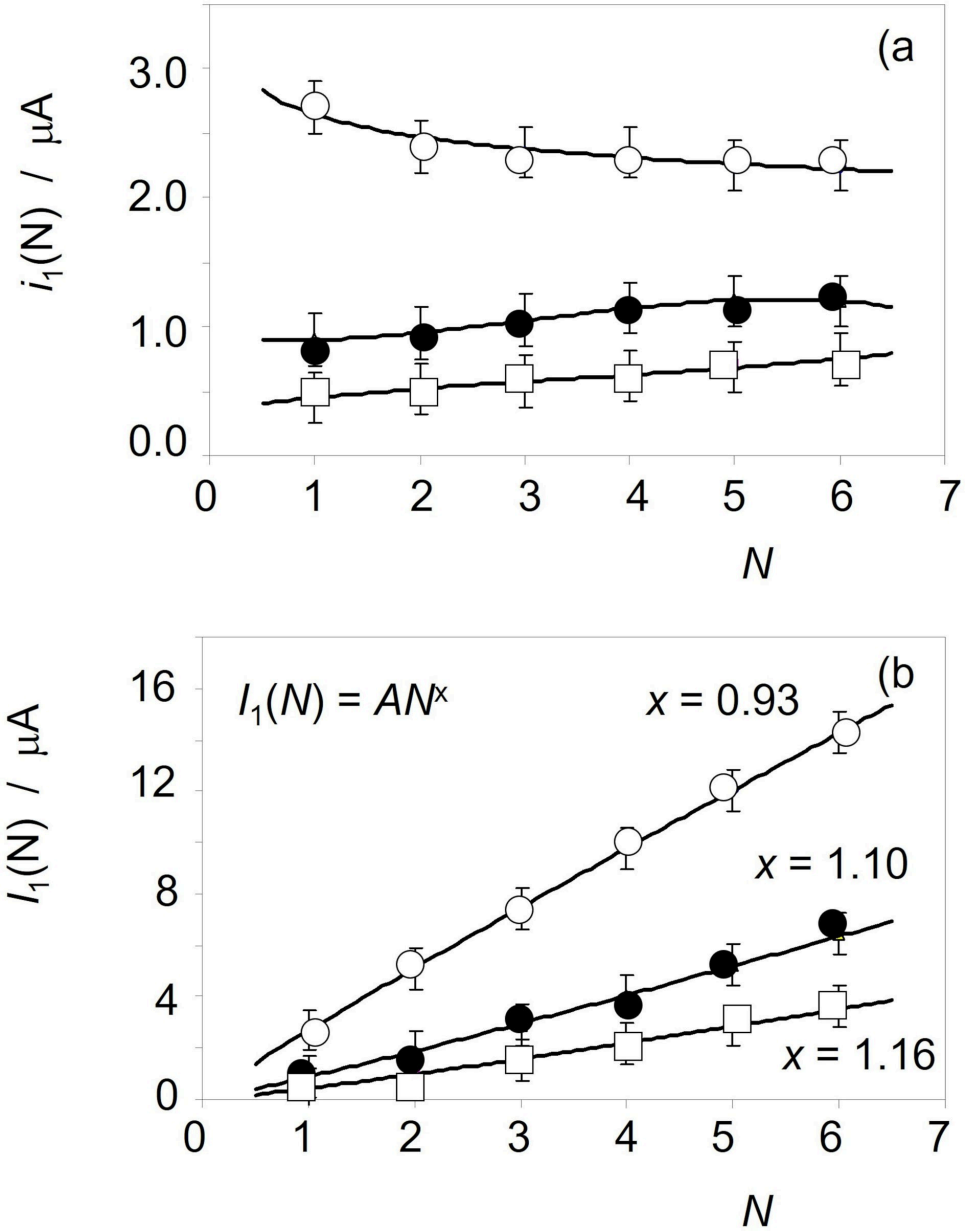
Variation of a) the peak current and b) the accumulated peak current of the process C_1_ with the scan number *N* for samples taken from a sword from the site of Bovec, Slovenia (Natural History Museum, Vienna, inv. no. 32738) dated back to the Ha A2 period. The continuous lines in a) correspond to the fit of the experimental data points to a polynomic function of degree 3, while the continuous lines in b) correspond to the fit of experimental data to a potential function of the exponent x taken with N as a continuous variable.

Figure 5a shows the variation of *i*_1_(*N*) with *N* for samples taken from three different areas of a sword from the site of Bovec, Slovenia (Natural History Museum, Vienna, inv. no. 32738). Since the net amount of sample adhered to the graphite electrode is different in each voltammetric experiment, the absolute values of the peak currents differ. Depending on the local conditions of the bronze surface, the values of *i*_1_(*N*) show different tendencies in successive potential scans. These can be taken, as already discussed,[^28^, ^29^, ^30^, ^31^, ^32^] as representative of the variation of the crystallinity and compactness of the cuprite responsible for the C_1_ peak in the successively layers of the patina flakes adhering to the graphite electrode.

Given the non‐uniformity of the *i*_1_(*N*) vs. *N* patterns, it is interesting to look at the accumulated peak currents, *I*_J_(*N*). This quantity, defined as the sum of the peak currents in the previous *N*‐scans, is representative of the cumulative charge passed after these potential scans. Assuming that the sheets are not completely delaminated, the depth *z*(*N*) reached at the *N* ^th^ scan is *z*(*N*)=Σ*δ*(*N*). Ideally, this will be the cumulative peak current determined for the *J*‐peak,

(14) $$I_{J}\;\left(N\right)=\sum i_{J}\left(N\right)=\;\sum g_{J}\delta \left(N\right)\rho_{J}\left(N\right)f_{J}\left(N\right)+b_{J}\left(N\right)$$

where *g*_J_=*knAh*_J_. For a patina of uniform composition and density, *z*(*N*) will be proportional to *I*_J_(*N*). If *δ*(*N*) and *ρ*(*N*) are equal in all scans (or, equivalently, their product remains constant), the cumulative peak current will be directly representative of the in‐depth variation of the mass fraction of the component J. Notice that, since in successive potential scans there is opportunity for oxidation of copper to Cu^2+^ (aq) and reduction of such ions in solution, the accumulated peak currents represent a more complex situation than that described by Eq. (14).

Despite these limitations, it is reasonable to assume that both *i*_J_(*N*) and *I*_J_(*N*) currents are representative of the variation with depth of the properties – namely chemical composition, crsytallinity, degree of hydration, compactness ‐ of the patina. The use of the accumulated peak currents facilitates data handling because they provide monotonically increasing values in successive scans. Typical data, again corresponding to three different points on a sword from the site of Bovec, Slovenia (Natural History Museum, Vienna inv. no. 32738), are shown in Figure 5b. Treating *N* as a continuous variable *I*_1_(*N*) and *I*_2_(*N*) can be fitted to potential functions of *N* of the type *I*_J_(*N*)∝*N* ^x^ close to *x*=1 with high values of the correlation coefficient (always higher than 0.9995). The differences in the *x* values can be attributed to the corrosion pattern in the different regions of the object and/or to the different depth reached in each sampling.

### Tenorite/cuprite ratio

The ratio between the peak currents (and the cumulative peak currents) for the processes C_2_ and C_1_, *i*_2_(*N*)/*i*_1_(*N*) (and *I*_2_(*N*)/*I*_1_(*N*)) can in principle be taken as representative of the tenorite/cuprite ratio at different depths in the patina. For dating purposes, however, the focus is on the tenorite/cuprite ratio, which is related to the ratio between the *I*_1_(*N*) and *I*_2_(*N*) signals. The general expression for this ratio is (Eq. 15,

(15) $$\frac{I_{2}\;\left(N\right)}{I_{1}\left(N\right)}=\;\frac{\sum g_{2}\delta \left(N\right)\sigma \left(N\right)f_{2}\left(N\right)+b_{2}\left(N\right)}{\sum g_{1}\delta \left(N\right)\rho \left(N\right)f_{1}\left(N\right)+b_{1}\left(N\right)}$$

Or, equivalently(Eq. 16)

where *G*_cup_, *G*_ten_, reflect the proportionality between the charges and the measured peak currents. Applying the formalism represented by Eqs. (3)‐(12), and eliminating *z*_N_ between Eqs. (7) and (11), we will obtain a potential relationship between *Q*_ten_(*N*) and *Q*_cup_(*N*) and, hence, between *I*_2_(*N*) and *I*_1_(*N*). This relationship will depend on the values of the exponents*α*, *β*, as well as on the values of the *ρ*_inn_, *ρ*_surf_, *σ*_inn_, *σ*_surf_ densities. In the simplest case of a linear variation of the cuprite and tenorite densities with dept, *Q*_ten_(*N*) will on *Q*_cup_(*N*) vary between two extreme linear tendencies (Eq. 17); for *z*_N_≪*z*_inn_,

(17) $$Q_{\mathrm{ten}}\left(N\right)\approx \frac{\sigma_{\mathrm{inn}}}{\rho_{\mathrm{inn}}}Q_{\mathrm{cup}}\left(N\right)\;$$

while for *z*_N_ approaching *z*_inn_ (Eq. 18,

(18) $$Q_{\mathrm{ten}}\left(N\right)\approx \left(\frac{\sigma_{\mathrm{inn}}}{\rho_{\mathrm{inn}}}\right)Q_{\mathrm{cup}}\left(N\right)+\sigma_{\mathrm{inn}}nFAz_{\mathrm{inn}}\left(\frac{\rho_{\mathrm{inn}}-\rho_{\mathrm{surf}}}{2}+\frac{\sigma_{\mathrm{surf}}-\sigma_{\mathrm{inn}}}{2}\right)$$

For our purposes, the relevant point to emphasize is that the values of the parameters *α*, *β*, *ρ*_inn_, *ρ*_surf_, *σ*_inn_, *σ*_surf_ will be dependent on the manufacturing type and corrosion history and age of the artifact. Accordingly, the differences in these parameters due to variations in manufacturing, age, etc. will be reflected in differences in the *Q*_ten_(*N*) vs. *Q*_cup_(*N*) and, ultimately, in differences in the *I*_2_(*N*) vs. *I*_1_(*N*) relationship.

### Comparison with experimental data

**Figure 6:**
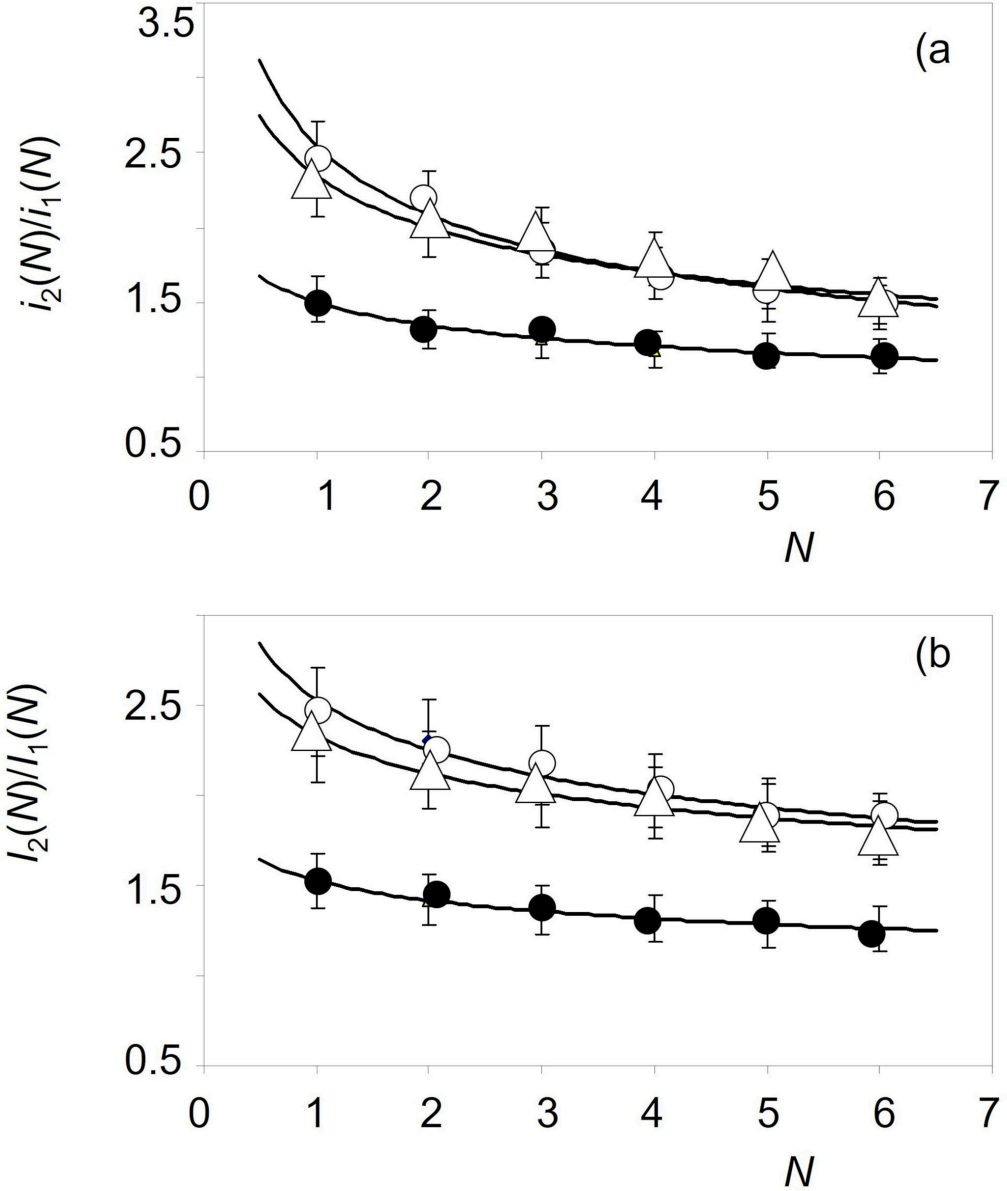
Variation of the: a) *i*_2_(*N*)/*i*_1_(*N*) and b) *I*_2_(*N*)/*I*_1_(*N*) ratios with the scan number *N* for samples taken from an Aldrans type sword with unknown site spot (Natural History Museum, Vienna, inv. no. 51251), dated back to the Ha A2 period. The continuous lines correspond to the fit of the experimental points to a potential function of the exponent *x* taken *N* as a continuous variable. Errors bars correspond to 10 % data scatter.

According to the previous results,[^28^, ^37^, ^38^, ^39^] the *I*_2_(1)/*I*_1_(1) ratio is representative of the outer composition of the patina. Since the aerobic oxidation of cuprite to tenorite is a thermodynamically favored process,^[28]^ this outer region should be enriched in tenorite relative to the inner patina layers. Figure 6 shows the variation of these ratios with the scan number *N* for samples taken from three areas of a sword of Type Aldrans, from an unknown find spot (Natural History Museum, Vienna, inv. no. 51251) dated back to the Ha A2 period. In all cases, the peak current ratios decrease monotonically with the scan number, consistent with the idea that the tenorite/cuprite ratio decreases from the outer surface to the inner regions of the corrosion layer.[^30^, ^31^, ^32^]

**Figure 7:**
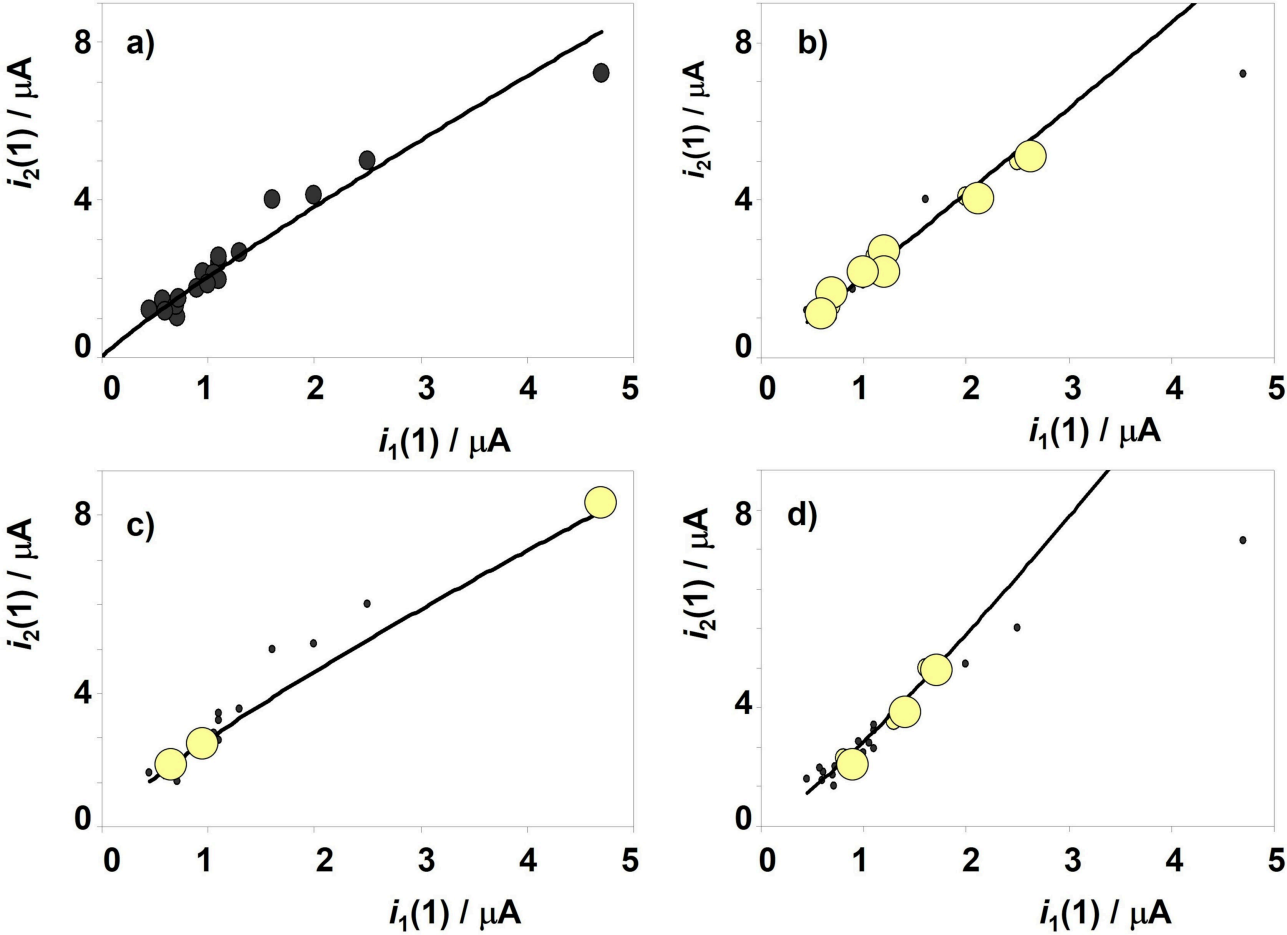
*i*_2_(1) vs. *i*_1_(1) plots for samples of Bz C and Bz D period in the Natural History Museum, Vienna. The whole data set (a) and separated data for samples from b) unknown find spot (inv. no. 18020) and Gusen, Austria (inv. no. 74150); c) Greiner Strudel, Austria (inv. no. 13878); d) Rovereto, Italy (inv. no. 74475). The continuous lines correspond to the fit of the experimental data poitns to a potential function of the exponent *x*. Error bars are omitted for simplicity.

As shown in several case studies,[^37^, ^38^, ^39^] the peak currents measured in the first scan are sensitive to minute variations in the composition and microstructure (and/or corrosion history) of the metal alloy. Figure 7 illustrates this type of data analysis. Here, Figure 7a shows the *i*_2_(1) vs. *i*_1_(1) plot for the entire data set taken for Bz C and Bz D samples from the Natural History Museum, Vienna. Experimental data can reasonably be fitted to a to a straight line passing by the origin (*i*_2_(1)=(1.56±0.10)*i*_1_(1)+0.51±0154; *r*=0.97) close to a potential function with exponent *x*=0.90±0.06 (ln*i*_2_(1)=(0.90±0.06)ln*i*_1_(1)+0.71±0.04; *r*=0.96). These variations are consistent with the expectances on applying Eq. (5) for both cuprite and tenorite signals.

In the general graph represented in Figure 7, however, it is possible to separate different trend curves for individual samples that follow such curved paths of this type, as can be seen in Figures 7b‐d. Interestingly, samples from Gusen, Austria, and an unknown find spot (Figure 7b), can be fitted to the same tendency graph (ln*i*_2_(1)=(1.02±0.06)ln*i*_1_(1)+0.72±0.03; *r*=0.990). The first one was clearly assigned to the Bz C2, period, while the second was assigned by archaeologist to the Bz C2–D period. The coincidence between these samples suggests that in the second sample can also be assigned to the Bz C2 period. In contrast, the sample Greiner Strudel sample (Figure 7c) from the same region and assigned to the Bz C period, shows a different trend curve (ln*i*_2_(1)=(0.85±0.11)ln*i*_1_(1)+0.63±0.10; *r*=0.992), thus suggesting a different production and/or corrosion history. Figure 7d shows the trend curve defined by one sample from Rovereto, Italy. (ln*i*_2_(1)=(1.2±0.2)ln*i*_1_(1)+0.76±0.08; *r*=0.98). This curve differs significantly from the above suggesting differences in the provenance and/or manufacturing procedure. Similar results were obtained for all other sample sets (see Supplementary Information, Figures S.4 to S.6).

### Chronological issues

**Figure 8:**
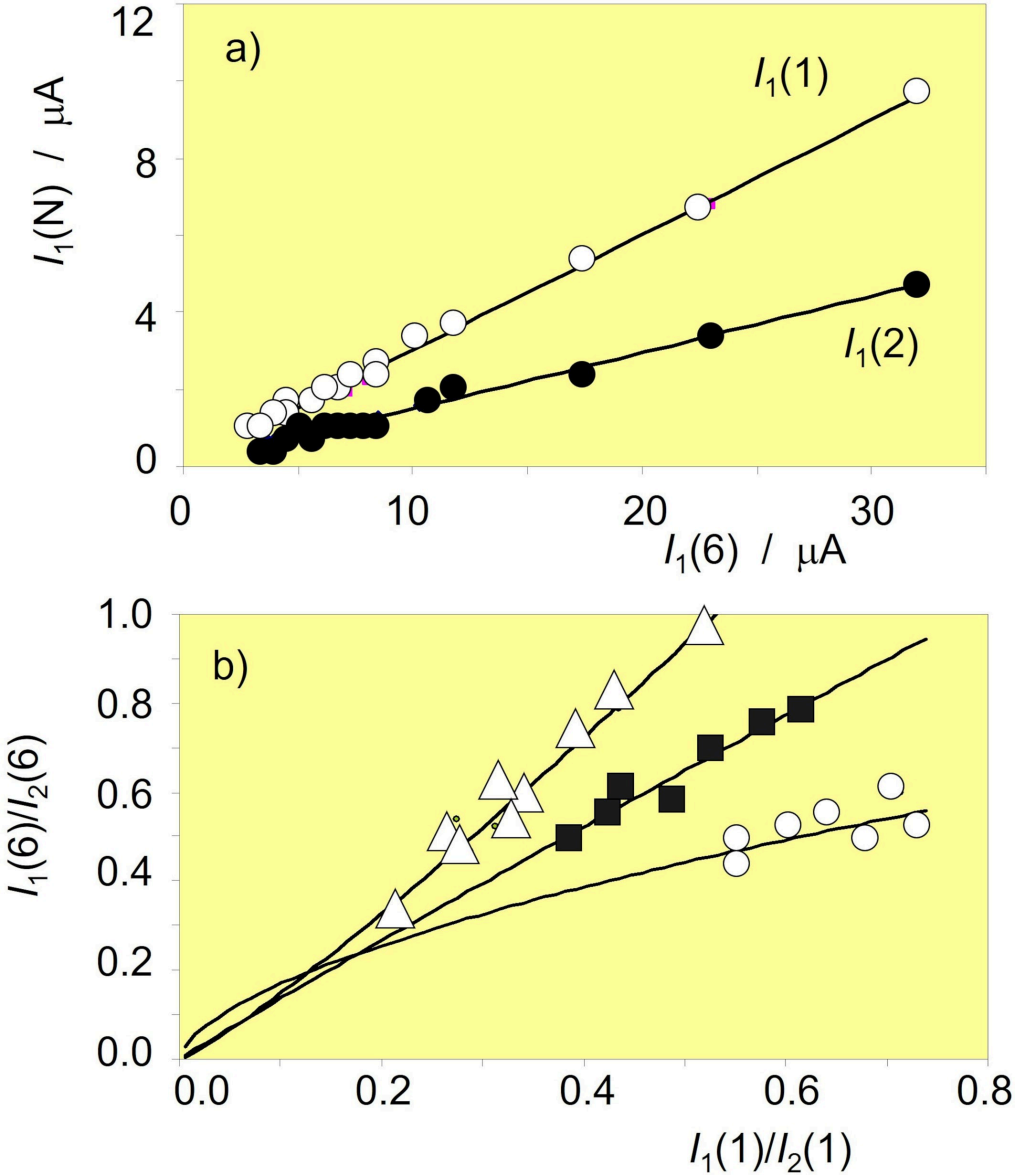
a) Variation of *I*_1_(1) and *I*_1_(2) vs. I_1_(6) for samples of Bz C period in the Natural History Museum, Vienna, using data in voltammograms such as in Figure 1. Error bars are omitted for clarity. b) Variation of the *I*_1_(6)/*I*_2_(6) ratio on the *I*_1_(1)/*I*_2_(1) ratio for two swords of BzC‐D period (circles, Natural History Museum, Vienna, inv. no.s 18020 and 74150), four 8th‐9th century CE rings (squares, University of Innsbruck, inv. no.s 13, 21, 140, and A6994) and two French coins of 1904 (triangles). Data points correspond to 2–3 three plicate measurements in different areas of the objects. Error bars are omitted for simplicity.

A second set of analytical criteria for grouping metal samples and establishing chronological issues can be derived by comparing the peak currents recorded in successive potential scans, now preferably using cumulative peak currents. Figure 8a shows the plots of *I*_1_(1) and *I*_1_(2) vs.*I*_1_(6) for 19 samples of BzC and BzD period from the Natural History Museum,Vienna. In both cases, satisfactory linearity was obtained (*I*_1_(1)=(0.299±0.004)*I*_1_(6)+(0.02±0.04), *r*=0.998; *I*_2_(1)=(0.146±0.003)*I*_1_(6)+(0.04±0.04), *r*=0.996) with intercepts close to zero. These features suggest that, despite the individual differences between samples, there is an essentially common variation in the corrosion pattern, reflected in the variation of the peak currents in successive potential scans. These data can be considered as consistent with the Eqs. (12) and (15)–(16) and, ultimately, with Eqs. (17)‐(18).

The possibility of age‐dependent voltammetric patterns despite the differences between individual samples, can be seen in Figure 8b, where the *I*_1_(6)/*I*_2_(6) ratio is plotted against the *I*_1_(1)/*I*_2_(1) one for two BzC‐D swords, four 8^th^‐9^th^ century CE rings and two French coins from 1904, in all cases superimposing the data point for 2–3 treplicate measurements in different areas of the objects. It can be seen that each series can be fitted to potential functions of the type *I*_1_(6)/*I*_2_(6)=*Y*[*I*_1_(1)/*I*_2_(1)]^y^ and, with a slightly lower correlation coefficient, to linear functions of the type *I*_1_(6)/*I*_2_(6)=SL[*I*_2_(1)/*I*_2_(1)]+OO. In these graphs, the *y*‐exponent and the slope SL increase on decreasing the age of the objects.

**Figure 9:**
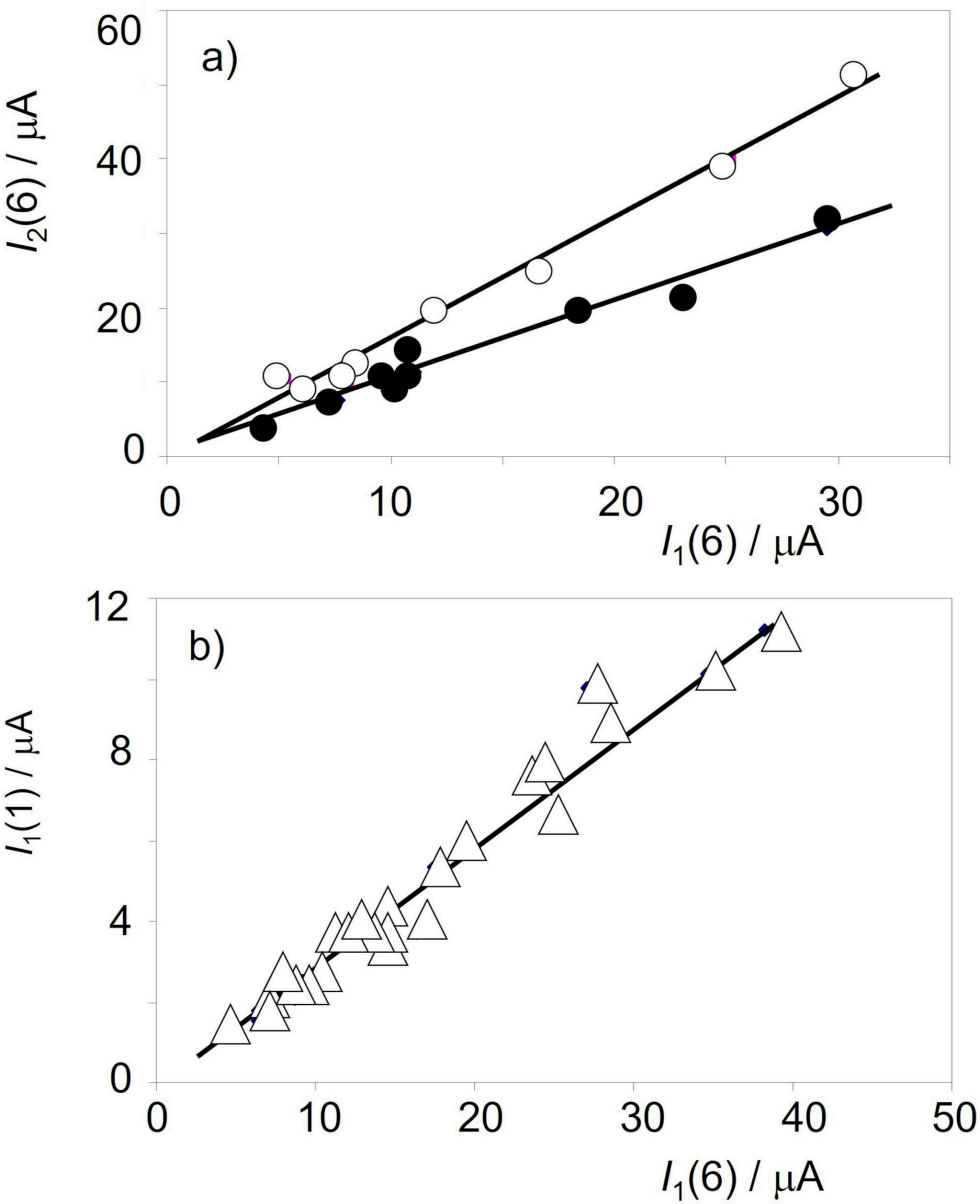
Variation of *I*_2_(6) vs. *I*_1_(6) for samples from the Russian sites of Koban (solid circles) and Chmi plus the Koban sample inv. no. 42186 (circles) from the Natural History Museum, Vienna, and b) variation of *I*_1_(1) vs. *I*_1_(6) for the Ha C period samples from the University of Innsbruck. The solid line corresponds to the linear fit of the experimental data points. Error bars are omitted for brevity.

Interestingly, multiple scan voltammetry data provide well‐defined criteria for discriminating between samples of different provenance. This is illustrated in Figure 9a which shows the variation of *I*_2_(6) vs. *I*_1_(6) for dagger samples from the sites of Koban and Chmi (Russia) from the Natural History Museum Vienna. These were assigned by archaeologist to a period between the Bronze to Early Iron age. It can seen in this figure that the data points from Koban, except for those for sample inv. no. 42186, fall on a well‐defined straight trend line that is clearly separated from the Chmi samples plus the Koban sample inv. no. 42186.

**Figure 10:**
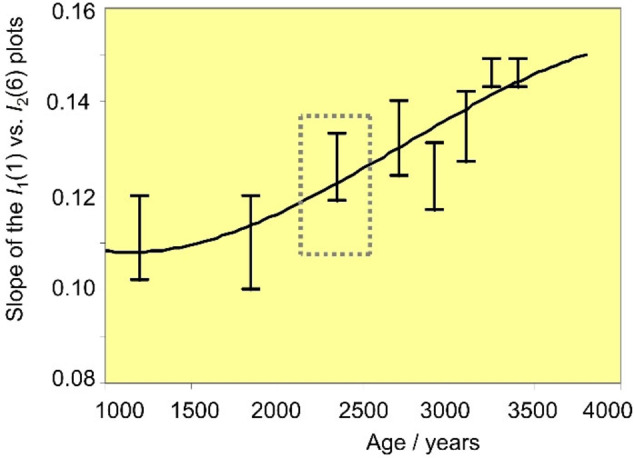
Scheme for Bronze Age chronology based on the values of the slope of the *I*_1_(1) on *I*_1_(6) linear plots such as in Figure 8b determined in repetitive voltammetry experiments (black segments) for samples of known age. The grey square corresponds to the age interval estimated for the finds derived from the archaeological sites of Koban and Chmi, Russia.

For age calibration purposes, the variation of *I*_1_(1) vs. *I*_1_(6) provides well‐defined linear responses as shown in Figure 9b for Ha C period samples from the University of Innsbruck. The slope of these plots appears to increase with the corrosion time, providing a variation with the age as shown in Figure 10. This is consistent with the previous set of considerations; the slope of the *I*_1_(1) to *I*_1_(6) plot can be considered as representative of the inverse of the gradient of tenorite concentration in the corrosion patina. Assuming that tenorite is formed from the primary patina of cuprite,[^37^, ^38^, ^39^] it can be expected that in ‘young’ patinas the gradient of tenorite will be greater (and the slope of the *I*_1_(1) vs. *I*_1_(6) plot will be lower) than in ‘old’ patinas.

The data in Figure 10 can be used to assign an age interval to the Koban and Chmi samples. From the slopes of the *I*_1_(1) vs. *I*_1_(6) plots (0.123±0.009 and 0.118±0.012, respectively), we obtain an age interval consistent with the archaeologist assignment.

## Conclusions

Voltammetric data for a series of copper/bronze archaeological artifacts including a set of samples from the European Bronze Age were obtained using the voltammetry of immobilized particles methodology on sample‐modified graphite electrodes in contact with aqueous acetate buffer at pH 4.75. The application of repetitive square wave voltammetry produces significant and systematic variations in the intensity of the signals corresponding to the reduction of cuprite and tenorite to metallic copper. Although these variations are sensitive to the composition, compactness, crystallinity and corrosion history of the artifacts, it is possible to establish regular patterns that characterize different types of samples.

The accumulated peak currents measured for cuprite and tenorite reduction signals vary monotonically the number of scans. These quantities can be satisfactorily related to potential and linear functions whose constitutive parameters can be used for age discrimination and age estimation purposes in homogeneous series of objects.

## Experimental

Samples were taken from ten archaeological copper and tin‐bronze artifacts from five different sites in Lower Austria from the MAMUZ Museum in Asparn an der Zaya (Austria); 38 objects and fragments form casting cakes from six sites (Micheldorf, Ansfelden‐Kremsdorf “Burgwiese”, Knopfsberg, Jochberg, Kitzbühel‐Römerweg, and Prutz‐Felsdach Steinegg) held by the Institute of Archaeologies, University of Innsbruck; 41 objects from sites different in Austria, the Czech Republic, Italy, Romania, Russia, Slovakia, Slovenia, and Ukraine held by the Natural History Museum in Vienna, as well as eleven coins from the collection of the Art History Museum, Vienna. The list of samples and their characteristics is given as a Supplementary Information, Tables S.1 to S.4.

Sampling was performed according to the standard VIMP procedure[^17^, ^18^, ^19^, ^20^] by pressing a graphite bar (type Faber‐Castell HB, diameter 2 mm) over the surface of the object. Planar regions with a uniform brownish or blackish hue were selected. Depending on the size of the object, 2–5 samples were taken from different parts of the surface. No previous scratching on the objects surface was made. The samples were collected in the museums under the supervision of the respective technical responsible and analyzed at the Department of Analytical Chemistry of the University of Valencia (Spain).

Voltammetric measurements were performed using a CH I920c potentiostat (Cambria Scientific, Llwynhendy, Llanelli UK) coupled to a conventional three‐electrode cell, with the sample modified graphite bar acting as the working electrode. The electrode assembly was completed with a platinum auxiliary electrode and an Ag/AgCl (3 M NaCl) reference electrode. A 0.25 M HAc/NaAc solution (Probus Reagents) at pH 4.75 was used as the supporting electrolyte being optionally deaerated by bubbling Ar.

The surface of several ‘modern’ coins was examined by field emission scanning electron microscopy (model S‐4800, Hitachi Ltd., Tokyo, Japan) operating at 20 kV. The microanalysis of the samples was carried out using a X‐ray microanalysis system (SEM/EDX). Sectioning of trenches and imaging of the coins in the trench were performed with a FIB‐FESEM Zeiss (Orsay Physics Kleindiek Oxford Instruments) model Auriga compact equipment; operating conditions: voltage, 30 kV, current intensity, 500 μA, and current of 20 nA in the FIB for generating the focused beam of Ga ions and a voltage of 3 kV in the FESEM for photographs. X‐ray line scanning was performed in the trench using an Oxford‐X Max X‐ray microanalysis system coupled to the FESEM controlled by Aztec software. A voltage of 20 kV and a working distance of 6–7 mm were used.

## Supporting Information

RList of studied objects containing relevant archaeological an compositional data; additional voltametric records and data processing, and calculations on the depth reached in voltametric experiments from SEM/EDX and VIMP data.

## Conflict of interest

The authors declare no conflict of interest.

1

## Supporting information

As a service to our authors and readers, this journal provides supporting information supplied by the authors. Such materials are peer reviewed and may be re‐organized for online delivery, but are not copy‐edited or typeset. Technical support issues arising from supporting information (other than missing files) should be addressed to the authors.

Supporting Information

## Data Availability

The data that support the findings of this study are available from the corresponding author upon reasonable request.
