# Supplementary material for: Electrochemical Approximation to Bronze Age Chronology via Multiple Scan Voltammetry
Source: ChemElectroChem. 2023 Nov 6;10(23):e202300405. doi: 10.1002/celc.202300405 (PMC10962692; doi:10.1002/celc.202300405)
Supplement: Supplementary file 1 — Supporting Information [file CELC-10-0-s001.pdf]

ChemElectroChem

Supporting Information

Electrochemical Approximation to Bronze Age Chronology via Multiple Scan Voltammetry

Antonio Doménech-Carbó,* Marianne Mödlinger, Laura Osete-Cortina, and María Teresa Doménech-Carbó

Figure S.1. Square wave voltammograms of a graphite electrode modified with sample in contact with air-saturated 0.25 M HAc/NaAc aqueous buffer at pH 4.75. Potential scan initiated at a) 0.45 V in the negative direction and b) Potential step increment 4 mV; square wave amplitude 25 mV; frequency 10 Hz.

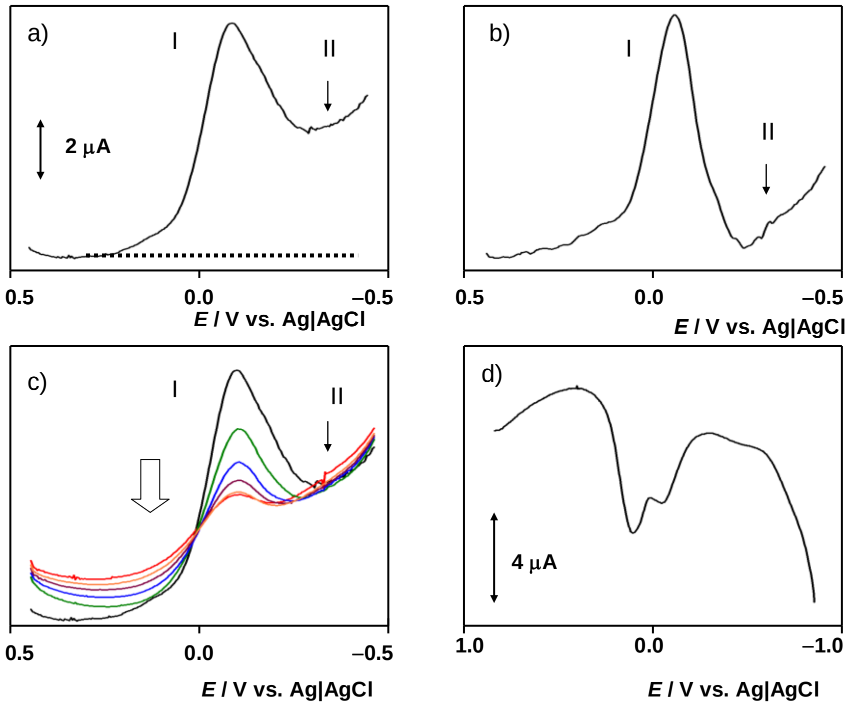

Figure S.2. Schematic representation of abrasive sampling with a graphite bar on the surface of a metallic object.

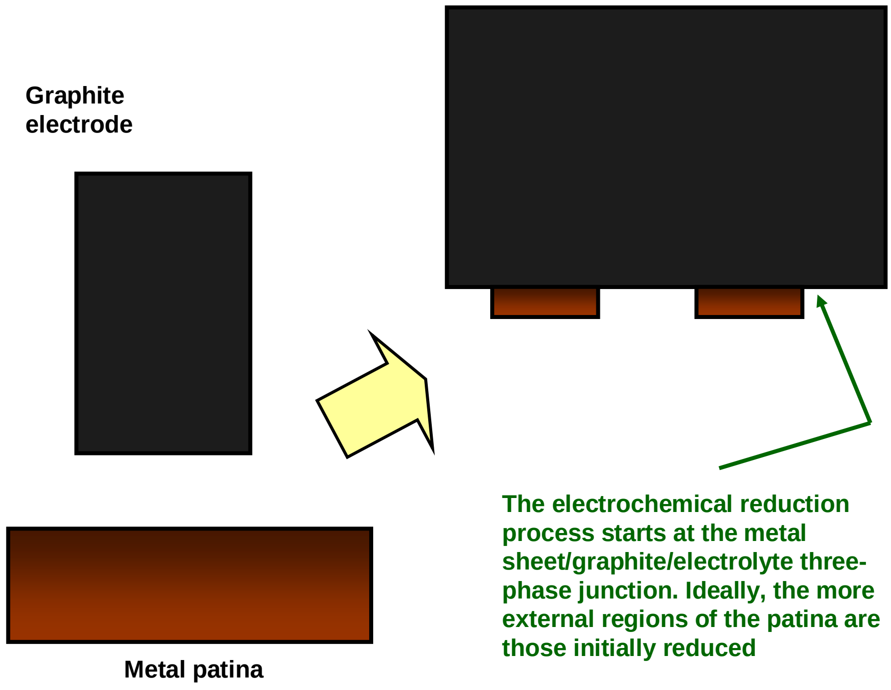

Figure S.3. Variation of the $i_2(N)/i_1(N)$ ratio on $I_1(N)$ for VIMP experiments in the conditions used in Figure 2 for the Cu-Zn coin whose SEM/EDX data are shown in Figure 4. Note the close similarity between the two curved paths. This is consistent with the idea that $I_1(N)$ is representative of the depth reached in the Nth scan and that the $i_2(N)/i_1(N)$ ratio is representative of the tenorite/cuprite molar ratio in the patina (see modeling section).

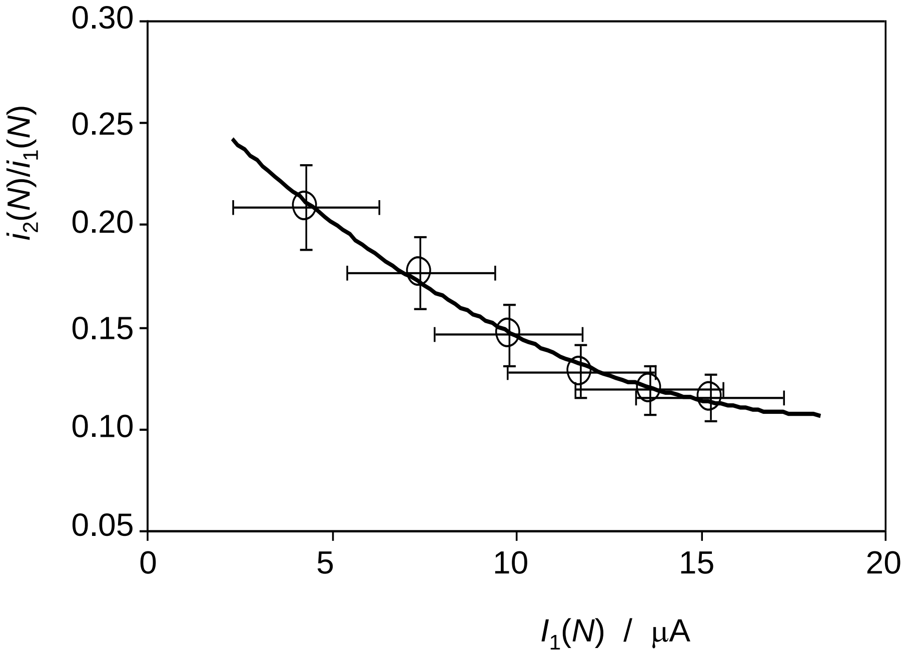

Figure S.4. $i_2(1)$ vs. $i_1(1)$ plots for samples assigned to the Ha A period from the Natural History Museum, Vienna. The continuous line corresponds to the fit of the experimental data points to a linear function. Error bars are omitted for brevity.

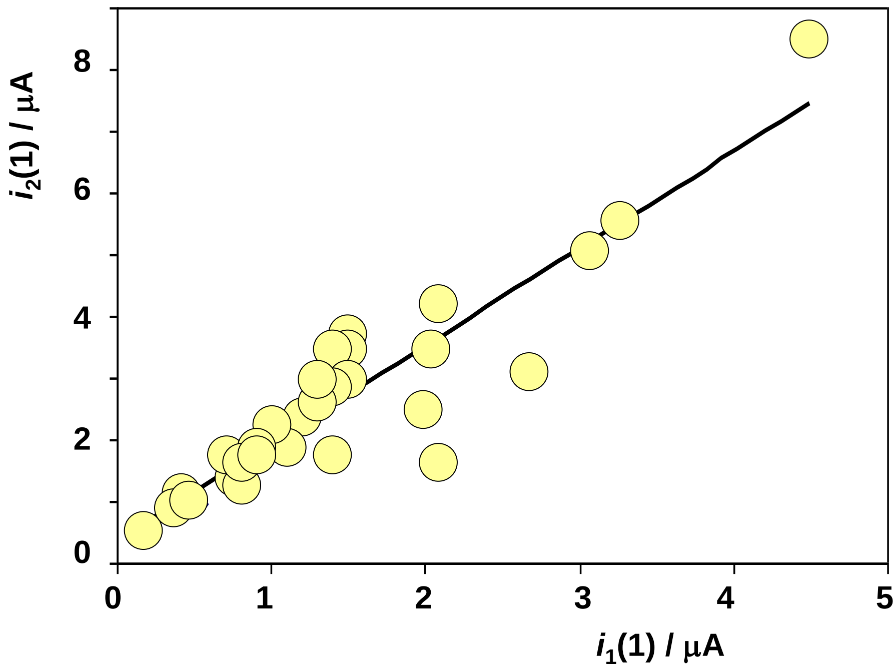

Figure S.5. $I_1(2)$ vs. $I_1(1)$ plots for samples of mediaeval age from the University of Innsbruck, Innsbruck. The continuous line corresponds to the fit of the experimental data points to a linear function. Error bars are omitted for brevity.

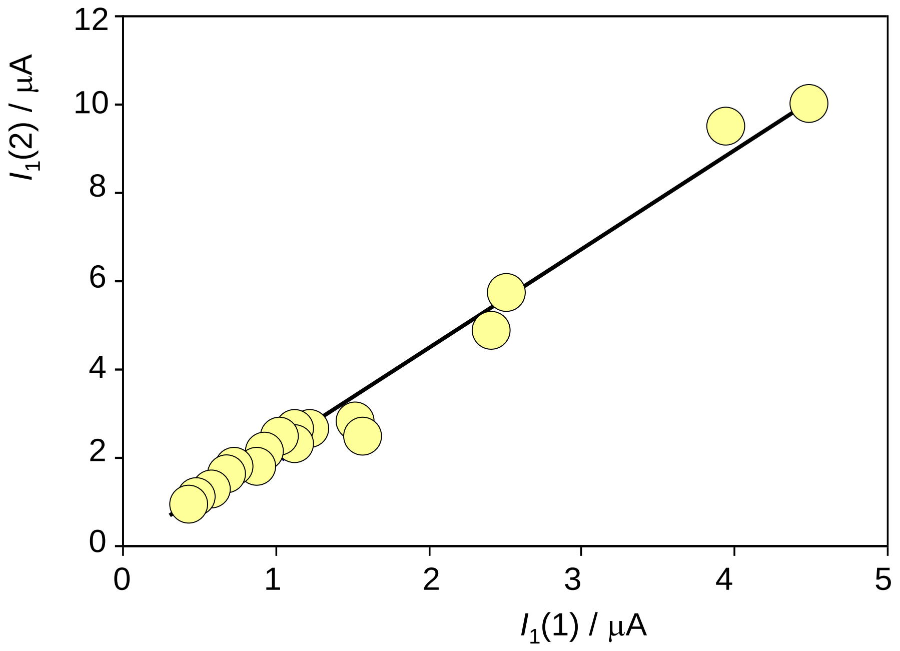

Figure S.6. Variation of $I_1(1)$ (circles) and $I_1(2)$ (solid circles) vs. $I_1(6)$ for samples assigned to the Ha C period from the University of Innsbruck, Innsbruck. The continuous lines correspond to the fit of the experimental data points to a linear function. Error bars are omitted for brevity.

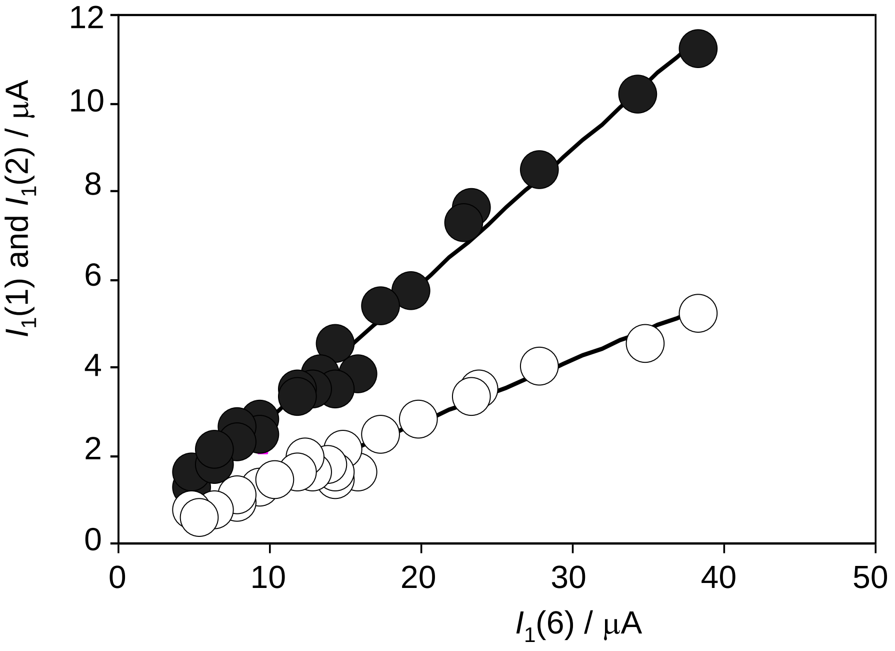

Depth calculations:

The SEM images in Figures 3 and 4a suggest that flakes of micrometric dimensions from the corrosion patina are transferred to the graphite electrodes during the sampling process. The area covered by these flakes can be estimated from the above images to be between 0.1% and 1% of the electrode area (0.0314 cm^2). Then, the area covered by the flakes ranges between 3.14 $\times 10^{-5}$ cm^2 and 3.14 $\times 10^{-4}$ cm^2. Assuming average flake dimensions of 0.5 × 0.5 × 0.5 μm, their averaged area is of 0.25 $\times 10^{-8}$ cm^2, so that the number of flakes can be estimated as between 1.26 $\times 10^{4}$ and 1.26 $\times 10^{5}$. The net volume of patina flakes on the electrode is then between 1.6 $\times 10^{-9}$ cm^3 and 1.6 $\times 10^{-8}$ cm^3. Assuming that the average density of the flakes is of 6 g cm^{-3} (intermediate between the densities of copper, and copper oxides), the corresponding mass will be between 1.0 $\times 10^{-8}$ g and 1.0 $\times 10^{-7}$ g. Assuming for the sake of simplicity that the flakes are composed of tenorite (molar mass 79.54 g mol^{-1}), this corresponds to 1.2 $\times 10^{-10}$ - 1.2 $\times 10^{-9}$ mol. The net charge corresponding to the two-electron reduction process involving with depletion of the flakes, will range between 23 and 230 μC.

In typical multiple-scan VIMP measurements, the charge passed, calculated from the area under the voltammetric peaks after 6 cathodic scans is typically between 5 and 50 μC. Assuming that the area of the flakes remains unchanged during the patina reduction processes, this means that the maximum depth achieved under our experimental conditions is approximately 25% of the patina thickness.

Table S.1 Archaeological and analytical information on the studied objects from the Mamuz Museum (Asparn an der Zaya).

**Inv. no.**	**Find spot**	**State**	**Context**	**Object**	**Relative chronology**	**Composition***
UF-22692.1272	Prigglitz-Gasteil	Austria	mining site	double-pointed “awl”	Ha B2-3	
UF-22692.1672	Prigglitz-Gasteil	Austria	mining site	double-pointed “awl”	Ha B2-3	
UF-22692.675	Prigglitz-Gasteil	Austria	mining site	casting cake	Ha B2-3	Cu (99.6)
UF-10,964	Prigglitz-Gasteil	Austria	mining site	Knife (reworked from a fragment)	Ha B2-3	
UF-22692.2188	Prigglitz-Gasteil	Austria	mining site	Knife (type Stillfried)	Ha B2-3	
UF-22692.912	Prigglitz-Gasteil	Austria	mining site	rod	Ha B2-3	
UF-19,452	Grünbach, Gelände	Austria	hilltop settlement	axe (type Haidach)	Ha A	
UF-9958	Prein an der Rax	Austria	smelting site	double-pointed “awl”	Ha B2-3	
UF-5098	Sieding	Austria	isolated find	axe (socketed) (special type)	Ha B	
[S041] = [S0780]	Reichenau, Kammerwandgrotte	Austria	cave	chisel	LBA?	

*Cu/Sn (circa 90/10) if not indicated

Table S.2 Archaeological and analytical information on the studied objects from the University of Innsbruck.

Inv. no.	Find spot	State	Context	Object	Relative chronology	Composition
M13	Notbergung Micheldorf	Austria	Grave 2	Ring fragment	Medieval	Unknown
M21	Notbergung Micheldorf	Austria	Grave 7	Ring	Medieval	Unknown
M27	Notbergung Micheldorf	Austria	Grave 8	Ring-headed needle/pin	Medieval	Unknown
M29	Notbergung Micheldorf	Austria	Grave 9	Fragment of a shield ring	Medieval	Unknown
M116	FMA Grfd. Micheldorf/Am Stein	Austria	Grave	Ring for the head?	Medieval	Unknown
M140	FMA Grfd. Micheldorf/Am Stein	Austria	Cemetery	Fibula	Medieval	Unknown
A6994	Micheldorf	Austria	Grave	ring	Medieval	Unknown
A6997	Micheldorf	Austria	Grave	ring	Medieval	Unknown
A6998	Micheldorf	Austria	Grave	Part of a belt?	Medieval	Unknown
A7103	Micheldorf	Austria	Grave	ring	Medieval	Unknown
A7105	Micheldorf	Austria	Grave	Part of a belt?	Medieval	Unknown
AK5139	Ansfelden-Kremsdorf “Burgwiese”	Austria	Unknown	arrowhead	LBA	Unknown
AK5648	Ansfelden-Kremsdorf “Burgwiese”	Austria	Unknown	knife	LBA	Unknown
K340	Prähist. Siedlungsareal Ruine Knopfsberg	Austria	Settlement	Fragment of casting cake	HaC	Cu/Ni/As/Sn/Sb/Pb (89/2/3/3/1/2)
K391	Prähist. Siedlungsareal Ruine Knopfsberg	Austria	Settlement	Rough copper fragment	HaC	Unknown
K392	Prähist. Siedlungsareal Ruine Knopfsberg	Austria	Settlement	Rough copper fragment	HaC	Cu/Ag/Sb (98/1/1)
K394	Prähist. Siedlungsareal Ruine Knopfsberg	Austria	Settlement	Bracelet fragment	HaC	Unknown
K396	Prähist. Siedlungsareal Ruine Knopfsberg	Austria	Settlement	Fragment of casting cake	HaC	Cu/Ag/Sb (91/1/7)
K436	Prähist. Siedlungsareal Knopfsberg	Austria	Settlement	Fragment of casting cake	HaC	Unknown
K439	Prähist. Siedlungsareal Knopfsberg	Austria	Settlement	Fragment of casting cake	HaC	Cu/Fe/Sb (94/1/3)
K440	Prähist. Siedlungsareal Knopfsberg	Austria	Settlement	Fragment of casting cake	HaC	Cu/Ni/Ag/Sb (94/1/1/2)
K443	Prähist. Siedlungsareal Ruine Knopfsberg	Austria	Settlement	Fragment of casting cake/rough copper fragment	HaC	Cu/Ag/Sb (90/1/8)
K460	Phähist. Siedlungsareal Knopfsberg	Austria	Settlement	sword/lance	HaC	Unknown
K463	Prähist. Siedlungsareal Knopfsberg	Austria	Settlement	Fragment of casting cake/rough copper fragment	HaC	Cu (100)
K464	Prähist. Siedlungsareal Knopfsberg	Austria	Settlement	Axe blade fragment	HaC	Cu/Sn/Sb (89/8/2)
K467	Prähist. Siedlungsareal Ruine Knopfsberg	Austria	Settlement	Fragment of casting cake/rough copper fragment	HaC	Unknown
K532	Prähist. Siedlungsareal Ruine Knopfsberg	Austria	Settlement	Fragment of casting cake	HaC	Cu/Fe/Sn (52/9/37)
K545	Prähist. Siedlungsareal Kropfsberg	Austria	Settlement	Pinhead?	HaC	Unknown
K846	Prähist. Siedlungsareal Kropfsberg	Austria	Settlement	Bronze bar fragment	HaC	Unknown
P76	Prospection Jochberg (Gamshag, Schützkogel, Teulfelssprung): Prähist Kupferbergbau	Austria	copper mining area	casting cake fragment	Transition from Middle to Late Bronze Age (13th century BCE)	Cu/Fe (98/1)
P32	Prospection Jochberg (Gamshag, Schützkogel, Teulfelssprung): Prähist Kupferbergbau	Austria	copper mining area	casting cake fragment	Transition from Middle to Late Bronze Age (13th century BCE)	Cu (99)

KB-22-2	Kitzbühel-Römerweg	Austria	Unknown	Sword	Late Bronze Age	Cu/Sn (86/12)
KB-22-1	Kitzbühel-Römerweg	Austria	Unknown	Casting cake	Late Bronze Age	Cu/Ag/Sb (95/1/3)
PS20-90	Prutz-Felsdach Steinegg	Austria	Unknown	Pin shaft	Likely Bronze Age	Unknown
PS20-147	Prutz-Felsdach Steinegg	Austria	Unknown	fibula?	Late antique (¿)	Unknown
P21-288	Prutz-Felsdach Steinegg	Austria	Unknown	?, restored	Late antique	Unknown
PS-1	Prutz Steinegg	Austria	Unknown	Coin, restored	Late antique	Unknown
PS-2	Prutz Steinegg	Austria	Unknown	Coin, restored	Late antique	Unknown

Table S.3 Archaeological and analytical information on the studied objects from the Natural History Museum (Vienna).

Inv. no.	Find spot	State	Context	Object	Relative chronology	Composition*
1926	Vulchovica, Ukraine	Ukraine	deposit	sword	Ha B1	
1930	Vulchovica, Ukraine	Ukraine	deposit	sword	Ha A2	
12638	Tscheraditz, Tschechien	Czech Republic	grave	sword	Ha A2	
13879	Grein, OÖ	Austria	water	sword	?	
18017	Velká, Slowakei	Slovakia	deposit	sword	Ha A2	
18020	unknown	unknown	unknown/single find	sword	Bz C2 – D	
32738	Bovec, Slowenien	Slovenia	unknown/single find	sword	Ha A2	
35011	unknown	unknown	unknown/single find	sword	Ha A	
35012	unknown	unknown	unknown/single find	sword	Bz B1	
35617	unknown	unknown	unknown/single find	sword	D – Ha A1	
47838	St. Pölten, NÖ	Austria	grave	sword	Ha A2	
51250	Năsăud, Rumänien	Romania	unknown/single find	sword	Ha A2	
51251	unknown	unknown	unknown	sword	Ha A2	
51258	Greiner Strudel, OÖ	Austria	water	sword	Ha A	
54054	Oberravelsbach, NÖ	Austria	unknown/single find	sword	Ha A2	
55955	Lorch, OÖ	Austria	water	sword	Bz D	
55994	Schlögen, OÖ	Austria	unknown/single find	sword	Ha A	
70652	Langmannersdorf, NÖ	Austria	grave	sword	Ha A1	
74150	Gusen, OÖ	Austria	grave	sword	Bz C2	
74275	Wimpassing, OÖ	Austria	water	sword	D – Ha A1	
74475	Rovereto, Prov. Trento, Italien	Italy	water	sword	Bz C	
85650	unknown	unknown	unknown/single find	sword	Bz D	
41.268 (A)	Koban, grave IV (juvenil, male?)	Russia	grave	dagger	MBA / LBA	Cu/Sn/Sb (89/10/0.6)
41.283 (B)	Koban, grave V (juvenil)	Russia	grave	dagger	Early Bronze Age	Cu/As (94/6)
41.389 (C)	Koban, grave XI (adult, male)	Russia	grave	dagger	MBA / LBA	Cu/As (93/7)
41.631 (D)	Koban, grave XVI (juvenil male)	Russia	grave	dagger	MBA / LBA	Cu/As/Sb (93/5/1)
41.788 (E)	Koban, grave XXIX (adult, male)	Russia	grave	dagger	MBA / LBA	Cu/Sn/Sb (82/17/1)
41.803 (F)	Koban, grave XXX (juvenil, male?)	Russia	grave	dagger	MBA / LBA	Cu/As/Sn/Pb (94/4/1/0.6)
41.927 (G)	Koban (?)	Russia	grave	dagger	MBA / LBA	Cu/As/Sn/Sb/Pb (92/6/0.7/1/0.7)
41.928 (H)	Koban (?)	Russia	grave	dagger	MBA / LBA	Cu/As/Sn/Sb (92/3/4.6/0.4)
42.186 (I)	Koban, grave III (adult, male)	Russia	grave	dagger	MBA / LBA	Cu/As/Sn/Sb (90/2/5/2/1)
42.466 (J)	Chmi (grave I)	Russia	grave	dagger	early LBA	Cu/Sn/Sb (91/9/0.6)
42.623 (K)	Chmi (?)	Russia	grave	dagger	early LBA	Cu/As (97/3)
47.633	Škocjan	Slovenia	deposit (cave)	helmet	HaC	
75.839	Škocjan	Slovenia	deposit (cave)	helmet	HaB	
75.839	Stetten	Austria	deposit	greave	HaA	
47636	Škocjan	Slovenia	deposit (cave)	helmet	HaC	Unknown
34776	Mahrersdorf	Austria	deposit	median-winged axe	Bz C – D	
34777 (2118)	Mahrersdorf	Austria	deposit	end-winged axe	Ha B1	Cu/Sn/Sb (91/7/1)
34777 (2120)	Mahrersdorf	Austria	deposit	end-winged axe	Ha B1	
34778	Mahrersdorf	Austria	deposit	end-winged axe	Ha B1	

34779 A	Mahrersdorf	Austria	deposit	socketed axe	Ha B1	Cu/As/Sb (94/1/3)
34779 B	Mahrersdorf	Austria	deposit	socketed axe	Ha B1	Cu/Sn/Sb (96/1/2)
34779 C	Mahrersdorf	Austria	deposit	socketed axe	Ha B1	Cu/As/Sb (95/1/3)
34780	Mahrersdorf	Austria	deposit	socketed axe	Ha B1	Cu/As/Sb (94/1/3)
34781	Mahrersdorf	Austria	deposit	socketed adze	Ha B1	
34783	Mahrersdorf	Austria	deposit	lancet-shaped chisel	Bz D – Ha B	
34784	Mahrersdorf	Austria	deposit	double-axe shaped ingot	Ha B1	Cu/Sn/Sb (94/3/2)

*Cu/Sn (circa 90/10) if not indicated

Table S.4 Archaeological and analytical information on the studied objects from the Art History Museum (Vienna).

Inv. no.	Find spot	State	Context	Object	Chronology	Composition
9/84	Bad Deutsch-Altenburg, Flur Mühläcker	Austria	excaved soil (Aushub)	coin	first third 4th century AD	Cu/Sn/Ag/Pb (86/2/3/9)
40/84	Bad Deutsch-Altenburg, Flur Mühläcker	Austria	soil with gravel	coin	2nd hald 2nd century AD	Cu/Zn/Pb (48/2/50)
59/84	Bad Deutsch-Altenburg, Flur Mühläcker	Austria	cloggy soil	coin	117-138 (Hadrian)	Cu/Zn (82/18)
61/84	Bad Deutsch-Altenburg, Flur Mühläcker	Austria	soil with gravel	coin	2nd third 2nd century AD	Cu/Sn/Zn/Pb (78/6/6/9)
71/84	Bad Deutsch-Altenburg, Flur Mühläcker	Austria	burnt charcoal	coin	2nd century	Cu/Zn/Pb (76/22/2)
106/84	Bad Deutsch-Altenburg, Flur Mühläcker	Austria	soil with gravel	coin	161-180 AD	Cu/Sn/Pb (73/3/24)
133/84	Bad Deutsch-Altenburg, Flur Mühläcker	Austria	soil with mortar	coin	mid 3rd century AD	Cu/Ag (73/27)
187/84	Bad Deutsch-Altenburg, Flur Mühläcker	Austria	soil, burnt soil	coin	2nd half 2nd century AD (Commodus)	Cu/Sn/Zn/Pb (52/4/2/42)
335/84	Bad Deutsch-Altenburg, Flur Mühläcker	Austria	humus	coin	1st half 1st century AD	Cu/Zn/Pb (58/3/39)
360/84	Bad Deutsch-Altenburg, Flur Mühläcker	Austria	burnt soil	coin	117-138 ? (Hadrian?)	Cu/Zn/Pb (81/14/6)
371/84	Bad Deutsch-Altenburg, Flur Mühläcker	Austria	loam under wooden floor	coin	first half 3rd century AD	Cu/Ag/Pb (57/41/2)
